# Marginal fit of titanium and zirconia abutments: Micro-CT analysis

**DOI:** 10.6026/973206300221809

**Published:** 2026-03-31

**Authors:** Harikrishna Modalavalasa, Katta Bhargavi, Ramesh Ampolu, Prachi Krishna Kant Pandey, Supriya Shukla, Gaurav Kumar Jha

**Affiliations:** 1 Department of Prosthodontics, GITAM Dental College & Hospital, Visakhapatnam, India; 2 Department of Prosthodontics, Drs. S & NR SIDS, Gannavaram, India; 3 Department of Prosthodontics, GITAM Dental College & Hospital, Visakhapatnam, India; 4 Private Practitioner, Prosthodontist & Implantologist, Mahavir Arogya Sansthan, Bihar, Patna, India; 5 Department of Prosthodontics, Manav Rachna Dental College, Faridabad, Haryana, India; 6 Private Practitioner, Oral and Maxillofacial Surgeon, Mahavir Arogya Sansthan, Patna, India

**Keywords:** Digital dentistry, computer-aided design and computer-aided manufacturing (CAD/CAM), micro-computed tomography (micro-CT), bacterial microleakage, implant abutments, marginal adaptation

## Abstract

The lack of high-resolution three-dimensional evidence comparing marginal fit and biological sealing between CAD/CAM titanium and zirconia abutments manufactured under identical digital workflows remains a critical gap in implant prosthodontics. Digital workflows enhance implant prosthodontic precision, yet differences in marginal fit between CAD/CAM titanium and zirconia abutments are unclear. Therefore, it is of interest to assess marginal adaptation, internal fit and bacterial microleakage of 60 digitally fabricated abutments using micro-CT and bacterial infiltration tests. Titanium abutments showed smaller marginal gaps (12.4 ± 3.8 µm) and higher internal contact (73.2 ± 8.4%) than zirconia (23.7 ± 6.2 µm; 58.9 ± 11.7%) (p < 0.001). Bacterial leakage was lower in titanium (13.3%) compared to zirconia (43.3%) (P < 0.05). Thus, CAD/CAM titanium abutments demonstrated superior marginal fit, internal adaptation and microbial sealing.

## Background:

The integration of digital workflows in implant prosthodontics has markedly improved clinical practice by enhancing diagnostic accuracy, precision of implant placement and consistency in prosthetic fabrication [1]. The application of computer-aided design and computer-aided manufacturing (CAD/CAM) technologies has enabled the fabrication of custom implant abutments with improved dimensional accuracy and more predictable emergence profiles when compared with prefabricated components [2]. Despite the widespread clinical adoption of CAD/CAM systems, there remains limited understanding of how different abutment materials perform when subjected to identical digital manufacturing conditions. Previous evidence has highlighted that abutment material plays a crucial role in determining clinical performance and long-term outcomes, even when standardized digital workflows are employed [3]. This underscores the need for controlled investigations that isolate material-related effects under uniform manufacturing parameters. Material selection is a key factor influencing the longevity and success of implant-supported restorations. Titanium abutments have traditionally been considered the gold standard due to their excellent biocompatibility, mechanical strength and corrosion resistance, with studies reporting predictable marginal adaptation and accurate implant-abutment interface fit [4].

Zirconia abutments, in contrast, have gained popularity primarily because of their favorable esthetic properties and improved peri- implant soft-tissue response, particularly in the anterior region [5]. However, comparative investigations have indicated that differences in wear behavior and interfacial adaptation may exist between titanium and zirconia abutments, potentially affecting the integrity of the implant-abutment interface [6]. In addition to mechanical considerations, biological sealing at the implant-abutment interface plays a critical role in maintaining peri-implant health. Marginal discrepancies may act as bacterial reservoirs, promoting microleakage, inflammation and peri-implant bone loss, yet limited evidence is available correlating digitally manufactured abutment accuracy with bacterial sealing performance under standardized CAD/CAM conditions [7]. Accurate evaluation of marginal and internal adaptation is therefore essential, as material properties and manufacturing processes influence the fit and performance of implant components [8] and conventional two-dimensional sectioning techniques inadequately represent the complex three-dimensional morphology of the implant- abutment interface. Micro-computed tomography (micro-CT) has emerged as a reliable, non-destructive technique for high-resolution, three- dimensional assessment of marginal and internal fit, allowing precise volumetric evaluation of interfacial gaps and contact areas. Therefore, it is of interest to compare the marginal adaptation, internal fit and bacterial sealing ability of CAD/CAM-fabricated titanium and zirconia abutments produced using identical digital workflows.

## Materials and Methods:

## Study design and ethical approval:

This *in vitro* comparative study was designed to eliminate confounding variables while focusing specifically on material-related differences in CAD/CAM abutment fabrication. All procedures were conducted in temperature and humidity-controlled laboratory environments to ensure measurement precision.

## Sample size calculation:

Sample size calculation was performed using SPSS software based on pilot study data showing mean marginal gap differences of 8µm between materials with standard deviation of 6µm. With α=0.05 and power=90%, a minimum of 23 specimens per group was required. The sample size was increased to 30 per group to account for potential specimen loss during micro-CT analysis and to enhance statistical power for subgroup analyses.

## Implant system and standardization:

A single implant system (Straumann Tissue Level, 4.1mm diameter, 10mm length, Basel, Switzerland) was selected to eliminate system- related variables. Sixty identical implant replicas were fabricated from the original implant using high-precision casting to ensure complete dimensional consistency. All replicas were verified for dimensional accuracy using coordinate measuring machine (CMM) analysis before study initiation.

## Digital workflow protocol:

## Intraoral scanning simulation:

A master cast containing a single implant with scan body was created to represent the clinical situation. Digital impressions were captured using a laboratory scanner (3Shape E4, Copenhagen, Denmark) with 5µm accuracy. The same scan body position was maintained for all acquisitions to ensure identical digital input data.

## CAD design protocol:

Custom abutments were designed using standardized parameters in CAD software (3 Shape Implant Studio):

[1] Emergence angle: 30 degrees from implant axis

[2] Gingival height: 3mm uniform

[3] Margin thickness: 0.8mm

[4] Anti-rotational feature: hexagonal connection

[5] Surface roughness: Ra <0.2 µm specification

Identical design files were created for both material groups, with only material assignment differing between groups. Design validation included dimensional verification and manufacturability analysis for both materials.

## CAM Manufacturing:

All specimens were manufactured using the same 5-axis milling machine (Datron D5, Mühltal, Germany) to eliminate equipment variables:

## Titanium group (Group Ti):

Grade 4 commercially pure titanium blanks (Straumann CARES, Basel, Switzerland)

[1] Tool path optimization for titanium machining

[2] Cutting speed: 15,000 RPM

[3] Feed rate: 1,500 mm/min

[4] Flood coolant throughout machining

## Zirconia group (Group Zr):

Pre-sintered yttria-stabilized zirconia blanks (Straumann CARES, Basel, Switzerland)

[1] Tool path optimization for zirconia machining

[2] Cutting speed: 20,000 RPM

[3] Feed rate: 800 mm/min

[4] Air cooling to prevent thermal shock

[5] Post-milling sintering according to manufacturer specifications

## Micro-CT analysis protocol:

## Scanning parameters:

High-resolution micro-CT scanning was performed using a dedicated system (SkyScan 1272, Bruker, Belgium) optimized for dental applications:

[1] Voxel size: 2 µm isotropic resolution

[2] Voltage: 80 kV (titanium), 100 kV (zirconia)

[3] Current: 125 µA

[4] Rotation step: 0.3 degrees

[5] Frame averaging: 3

[6] Random movement: 20

Each abutment was scanned while seated on its corresponding implant replica with standardized insertion torque (35 Ncm) applied using a digital torque meter.

## Image reconstruction and analysis:

Micro-CT data reconstruction was performed using NRecon software (Bruker) with optimized parameters for each material. Three-dimensional analysis was conducted using CTAn and CTVol software packages with custom analysis protocols:

## Marginal gap analysis:

Perpendicular distance measurements at 36 equally spaced points around the abutment-implant interface. Statistical descriptors included mean, maximum and standard deviation of gap dimensions.

## Internal adaptation assessment:

Volumetric analysis of the interface space between abutment and implant internal surfaces. Contact area percentage was calculated as the ratio of areas with gaps <5 µm to total interface area.

## Void volume quantification:

Three-dimensional volumetric measurement of all spaces between abutment and implant surfaces, providing comprehensive assessment of fit quality.

## Bacterial microleakage testing:

## Bacterial strain and culture conditions:

Aggregatibacter actinomycetemcomitans (ATCC 43718) was selected as the test organism due to its association with peri-implant disease and appropriate size for microleakage detection. Bacterial cultures were maintained in brain heart infusion broth at 37°C in 5% CO2 atmosphere.

## Microleakage test setup:

A dual-chamber model was developed to assess bacterial penetration through the abutment-implant interface:

[1] Upper Chamber: Contains bacterial suspension (10^8 CFU/mL) surrounding the abutment

[2] Lower Chamber: Sterile medium for bacterial detection

[3] Connection: Abutment-implant assembly forms the only pathway between chambers

Specimens were sterilized using gamma irradiation and assembled under sterile conditions. Abutments were torqued to 35 Ncm using sterile technique.

## Microleakage assessment protocol:

Daily monitoring was performed for 14 days to detect bacterial penetration:

[1] Visual assessment for turbidity in lower chamber

[2] pH indicator color change detection

[3] Quantitative bacterial counts using colony forming unit (CFU) enumeration

[4] Time-to-penetration recording for positive specimens

## Surface characterization:

## Surface roughness analysis:

Surface roughness of all abutment surfaces was measured using optical profilometry (Olympus LEXT OLS4000):

[1] Measurement area: 50µm x 50µm at implant interface

[2] Parameters: Ra (arithmetic mean roughness), Rz (mean roughness depth)

Five measurements per specimen averaged for final values.

## Surface morphology assessment:

Scanning electron microscopy (SEM) analysis was performed on representative specimens:

[1] Acceleration voltage: 15 kV

[2] Working distance: 10mm

[3] Magnifications: 100x, 500x, 2000x, 5000x

Focus on machining marks and surface irregularities.

## Statistical analysis:

Statistical analysis was performed using R software (version 4.3.2) with α=0.05 significance level. Data normality was assessed using Shapiro-Wilk tests and Q-Q plots. Continuous variables were compared using independent t-tests or Mann-Whitney U tests based on distribution characteristics. Categorical variables were analyzed using Fisher's exact test. Multiple linear regression analysis was performed to identify predictors of marginal gap dimensions and bacterial penetration. Correlation analysis examined relationships between micro-CT measurements and bacterial microleakage outcomes. Inter-examiner reliability was assessed using intraclass correlation coefficients (ICC) for all measurements. Repeated measurements on 20% of specimens were performed to assess intra-examiner reliability.

## Results:

All sixty abutments were successfully fabricated according to the prescribed CAD/CAM protocols with no visible defects. Coordinate measuring machine (CMM) verification confirmed dimensional accuracy within a 10 µm tolerance for both groups. The mean manufacturing time was 45 ± 8 minutes for titanium abutments and 52 ± 12 minutes for zirconia abutments, the latter being longer due to the additional sintering process. No tool wear or failure was observed during milling (Table 1 - see PDF). Micro-CT analysis revealed significant differences in marginal and internal adaptation between the two materials. Titanium abutments demonstrated smaller mean marginal gaps (12.4 ± 3.8 µm) compared to zirconia (23.7 ± 6.2 µm, p < 0.001), as shown in (Table 2 - see PDF). Maximum gap values were also higher in zirconia (47.3 ± 12.1 µm) than in titanium (28.7 ± 8.2 µm, p < 0.001), indicating localized discrepancies. Titanium abutments exhibited greater contact area (73.2 ± 8.4%) than zirconia (58.9 ± 11.7%, p < 0.001). Gap distribution analysis showed that 56.8% of titanium interfaces were within 0-10 µm, compared with 31.2% for zirconia, confirming better adaptation in titanium abutments. Volumetric analysis demonstrated a 2.3-fold increase in void volume for zirconia abutments (0.201 ± 0.067 mm^3^) compared to titanium (0.087 ± 0.024 mm^3^, p < 0.001), indicating less uniform interface contact (Table 3 - see PDF). Titanium abutments displayed smaller, isolated voids, whereas zirconia abutments exhibited interconnected void networks. Bacterial microleakage testing revealed significantly greater bacterial penetration in zirconia specimens. Thirteen of thirty zirconia abutments (43.3%) showed penetration, compared to only four of thirty titanium abutments (13.3%), with an odds ratio of 5.1 (95% CI: 1.4-18.7, p < 0.05). The mean time to bacterial penetration was shorter for zirconia (4.7 ± 2.8 days) than for titanium (9.8 ± 3.2 days, p < 0.05). Logistic regression analysis indicated that every 10 µm increase in mean marginal gap increased the likelihood of bacterial penetration by 2.8-fold (p < 0.01). These findings are summarized in (Table 4 - see PDF). Surface characterization demonstrated significantly higher roughness in zirconia abutments, correlating with greater marginal gaps and bacterial leakage (Table 5 - see PDF). The arithmetic mean roughness (Ra) was 0.18 ± 0.04 µm for titanium and 0.31 ± 0.08 µm for zirconia (p < 0.001), while the mean roughness depth (Rz) was 1.24 ± 0.28 µm and 2.17 ± 0.52 µm, respectively. SEM analysis revealed smooth, consistent titanium surfaces with shallow striations, whereas zirconia displayed irregular textures with grain pullout and micro-cracks in 23% of specimens. Regression modeling identified surface roughness as a significant predictor of marginal gap formation for both materials, with stronger correlation in zirconia (R^2^ = 0.58) compared to titanium (R^2^ = 0.34). Logistic regression confirmed that material type (zirconia, OR = 5.1, p < 0.05), marginal gap (OR = 1.28 per µm, p < 0.001) and surface roughness (OR = 2.3 per 0.1 µm Ra, p < 0.05) were significant predictors of bacterial leakage, with the predictive model achieving 82.4% accuracy (AUC = 0.89). Measurement reliability was excellent across all parameters, as shown in (Table 6 - see PDF). Intraclass correlation coefficients (ICC) were 0.94 for marginal gap and 0.91 for internal adaptation, while bacterial penetration assessment showed near-perfect agreement (κ = 0.96). Coefficients of variation ranged between 3.2% and 5.8%, confirming high reproducibility and precision of measurements.

## Discussion:

The present study demonstrated that CAD/CAM-fabricated titanium abutments exhibited significantly smaller marginal gaps and lower bacterial penetration rates than zirconia abutments when produced using identical digital manufacturing protocols. These findings are consistent with previous three-dimensional evaluations showing that material properties substantially influence the accuracy and marginal adaptation of digitally fabricated implant abutments, even when CAD/CAM workflows are standardized [9].

The superior marginal and internal adaptation observed in titanium abutments can be explained by their favorable machinability and ductile behavior during milling. Evidence has shown that titanium allows smoother cutting dynamics and improved surface quality during machining [10], resulting in more precise reproduction of digitally designed interfaces. In contrast, zirconia's inherent brittleness predisposes it to micro-chipping and surface defects during machining, which may compromise marginal accuracy. Furthermore, post-sintering shrinkage associated with zirconia introduces dimensional distortion that is difficult to fully compensate for digitally, leading to increased discrepancies at the implant-abutment interface [11]. The biological relevance of these mechanical differences was confirmed by the bacterial microleakage findings. Zirconia abutments demonstrated significantly higher bacterial penetration rates and shorter time-to-leakage compared with titanium abutments. Prior investigations have demonstrated a direct relationship between marginal misfit and bacterial microleakage at the implant- abutment interface, indicating that even minimal discrepancies can permit bacterial penetration [12]. Reduced interfacial voids and smoother surface characteristics have also been associated with improved marginal and internal adaptation at the implant-abutment interface, as demonstrated by three-dimensional micro-CT analyses [1]. Additionally, improved interface integrity has been linked to enhanced prosthetic stability and clinical performance, as well as reduced microbial ingress and more favorable peri-implant tissue responses over time [14, 15]. Micro-computed tomography provided a comprehensive three-dimensional assessment of interface morphology in the present study. Titanium abutments displayed predominantly isolated voids and a higher percentage of contact area, whereas zirconia abutments exhibited larger and more interconnected void networks. Previous studies have emphasized that interconnected voids may facilitate bacterial migration and compromise decontamination efforts, thereby increasing the risk of peri-implant disease [16]. Moreover, increased contact area at the implant-abutment interface has been associated with improved stress distribution and enhanced mechanical stability of the implant-abutment connection [17, 18, 19, 20, 21- 22]. From a clinical perspective, these findings suggest that titanium abutments should be preferred in situations where marginal integrity and bacterial sealing are critical, particularly in subgingival or high-risk clinical scenarios. Zirconia abutments remain a valuable option in esthetically demanding regions; however, their use necessitates meticulous fit verification and long-term maintenance to minimize microleakage-related complications [23, 24, 25-26]. Future research should focus on optimizing material- specific CAD/CAM parameters, improving compensation strategies for zirconia sintering behavior and exploring surface modification approaches aimed at enhancing the precision and biological sealing of ceramic abutments.

## Conclusion:

We show that CAD/CAM titanium abutments is better than zirconia in manufacturing precision and bacterial sealing, showing superior marginal adaptation, internal fit and biological performance. Titanium's machinability enables more consistent fabrication and reduced bacterial leakage, underscoring the need for material-specific digital protocols and clinical validation.

## Advancement to knowledge:

This study provides the first high-resolution (2 µm voxel) three-dimensional micro-CT comparison of CAD/CAM titanium and zirconia abutments fabricated under identical digital workflows, eliminating manufacturing-related confounders. Unlike previous investigations that independently evaluated marginal fit or bacterial leakage, this study establishes a direct quantitative relationship between marginal gap dimensions, surface roughness and bacterial microleakage risk. The findings introduce a mechanistic model linking material machinability, interfacial adaptation and biological sealing, thereby offering clinically translatable evidence for material selection in digitally fabricated implant abutments.

## Funding statement:

Nil
